# Incidence and Treatment Strategy of Lateral Meniscus Posterior Root Tears and Ramp Lesions Identified During Isolated ACL Reconstructions: Report From a Nationwide Knee Ligament Register

**DOI:** 10.1177/23259671251399817

**Published:** 2025-12-23

**Authors:** Håvard Visnes, Andreas Persson, Anne Marie Fenstad, Eivind Inderhaug

**Affiliations:** † The Norwegian Knee Ligament Register, Department of Orthopedic Surgery, Haukeland University Hospital, Bergen, Norway; ‡ Oslo Sports Trauma Research Center, Norwegian School of Sports Sciences, Oslo, Norway; § Department of Orthopedics, Sorlandet Hospital Kristiansand, Kristiansand, Norway; ‖ Department of Orthopaedic Surgery, Oslo University Hospital, Oslo, Norway; ¶ Sports Traumatology and Arthroscopy Research Group, University of Bergen, Bergen, Norway; # Haukeland University Hospital, Bergen, Norway; Investigation performed at Norwegian Knee Ligament Register, Bergen, Norway

**Keywords:** ACL reconstruction, meniscus, ramp lesion, lateral meniscus posterior root tear, epidemiology

## Abstract

**Background:**

Meniscus repair is increasingly common during primary anterior cruciate ligament reconstruction (ACLR), particularly because of an increased focus on the severity of untreated lateral meniscus posterior root tears (LMPRTs) and ramp lesions. However, the incidence and current treatment strategies for these injuries across a larger population remain unclear as previous reports come from selective cohort studies.

**Purpose:**

To report on the incidence of LMPRTs and ramp lesions in primary ACLR in Norway, compare patient- and activity-related characteristics in those with and without these injuries, and describe current treatment strategies.

**Study Design:**

Cross-sectional study; Level of evidence, 3.

**Methods:**

Data from the Norwegian Knee Ligament Register (2018-2023) were analyzed. The study included patients undergoing isolated ACLR. Sex, age, weight, preoperative Knee injury and Osteoarthritis Outcome Score (KOOS) scores, sports participation, and meniscus treatment strategy were considered.

**Results:**

Of 5309 ACLR knees, 68% (n = 3605) had 1 or more recorded meniscus injuries, while LMPRTs and ramp lesions were seen in 10% and 7.8%, respectively. The ramp lesion tears were more frequently seen in younger patients (*P* < .001) and resulted in lower preoperative KOOS scores than in the LMPRT group, which comprised older patients exhibiting reduced preoperative KOOS scores. Winter sports exhibited the highest sport-specific incidence of LMPRTs (14.3%) and ramp lesions (14.2%). LMPRT treatment predominantly involved transtibial suturing with cortical fixation, while ramp lesion management was split between all-inside and other suturing techniques (including repairs through a posteromedial portal).

**Conclusion:**

This first nationwide registry study identified a high incidence of concomitant meniscus tears in ACLR, with incidences of LMPRTs and ramp lesions of 10.0% and 7.8%, respectively. Patients with ramp lesions were significantly younger than both those with LMPRTs and those without meniscus injuries. Treatment for LMPRTs predominantly involved transtibial sutures with cortical fixation, while treatment for ramp lesions was divided between all-inside suturing techniques and other suturing methods (including repairs through a posteromedial portal).

Meniscus repair has gained popularity, especially in anterior cruciate ligament reconstruction (ACLR) surgery.^39^ Data from anterior cruciate ligament (ACL) registries indicate that meniscus tears are present in 47% to 63% of patients with ACL injuries.^2,22^ Over the past 15 years, attention to the detrimental effects of meniscus removal and advancements in meniscus repair techniques, particularly regarding root tears and ramp lesions, have significantly influenced clinical practice.^10,30^

The lateral meniscus posterior root tear (LMPRT) is defined as a radial or longitudinal tear within 1 cm of the posterior root insertion site—or an injury to the meniscotibial ligaments.^4^ It has been reported to be present in 7% to 12% of knees with ACL injuries.^4,5,8,11,15,37,51^ A ramp lesion is defined as a medial meniscus tear involving the peripheral attachment of the posterior horn of the medial meniscus—almost exclusively associated with ACL injury.^13^ Although these lesions were previously underrecognized, the incidence of medial meniscus ramp lesions can be approximately 17% in patients who undergo ACLR and may be as high as 41% in certain subgroups.^12,40^

The possible clinical implications of untreated ramp lesions and/or LMPRTs include residual anterior translation and rotatory instability—potentially increasing the risk of early failure after ACLR.^3,18,24,46^ Treatment goals for both LMPRT and ramp lesion repair are therefore to normalize knee biomechanics to secure ACL graft integrity and to further prevent premature osteoarthritis development. There is no clear treatment algorithm for these injuries, with recommendations varying from nonoperative management to a range of surgical options. Although arthroscopic partial resection is commonly reported, preserving the menisci with a range of repair techniques is currently the preferred approach.^10,35,54^

The Norwegian Knee Ligament Register (NKLR) was established in 2004 as the world's first ACL register.^20^ Although meniscus lesions and surgeries have been registered since the NKLR's inception, these “novel” categories of meniscus injuries (LMPRT and ramp lesion) were not included before 2018. Since then, variables that describe the morphology of the meniscus tear—as well as its treatment strategy—have been reported. To our knowledge, there has been no national registry report on detailed meniscus injuries in association with ACL surgery.

Therefore, the aims of the current study are the following:

1. To determine the incidence of LMPRTs and ramp lesions observed during primary ACL reconstruction surgeries in Norway
2. To compare the patient- and sports-related factors of those with LMPRTs and ramp lesions to those with isolated ACL injuries
3. To outline the current treatment strategies for LMPRTs and ramp lesions

## Methods

This study was conducted in accordance with the Strengthening the Reporting of Observational Studies in Epidemiology checklist.^50^ It is a retrospective analysis of prospectively collected data from the NKLR. Since its inception in 2004, the NKLR has gathered information on all ACLRs performed from both public and private hospitals in Norway. Each year, around 2000 ACLRs are reported to the NKLR, and it contains high-validity data with around 85% to 90% coverage for primary ACLR across the country.^20,34^ Surgeons report surgical details and intraoperative findings to the register immediately after surgery, and the patients complete preoperative Knee injury and Osteoarthritis Outcome Score (KOOS). In 2018, a digital reporting system was introduced that included higher granularity on meniscus injuries, specifically details about LMPRT and ramp lesions.

### Study Population

**Figure 1.**
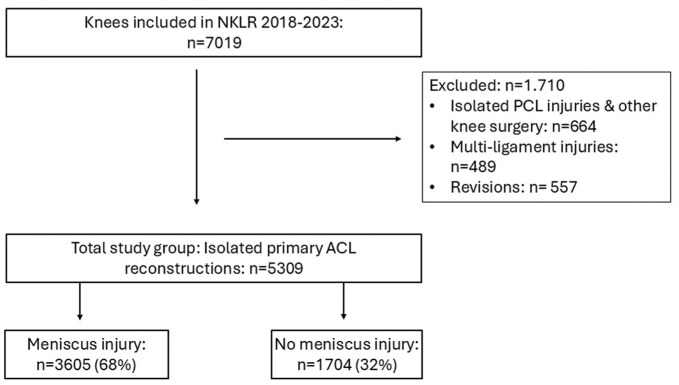
Flowchart of patient inclusion. ACL, anterior cruciate ligament; NKLR, Norwegian Knee Ligament Register; PCL, posterior cruciate ligament.

All patients registered with primary ACLR in the NKLR between January 1, 2018, and December 31, 2023, with a digital report, were eligible for inclusion (Figure 1). Isolated posterior cruciate ligament, other knee surgery, multiligament injuries, and revisions were excluded. However, patients with nonoperatively treated medial collateral ligament injuries were included.

### Variables and Outcome Measures

The following variables were extracted from the NKLR and included in the analyses:

*Patient-specific data:* age, sex, height (cm), weight (kg), activity at the time of injury, level of activity (level 1: pivoting sport [eg, soccer, handball, basketball and similar], level 2: less pivoting sport [eg, racket sports, alpine skiing, snowboarding, gymnastics, aerobics], level 3: no pivoting sport [eg, running, cross-country skiing, weightlifting], level 4: minimal physical activity/normal activities of daily living), date of injury, date of primary reconstruction, previous surgery to index knee or contralateral knee, and preoperative KOOS scores.

**Figure 2.**
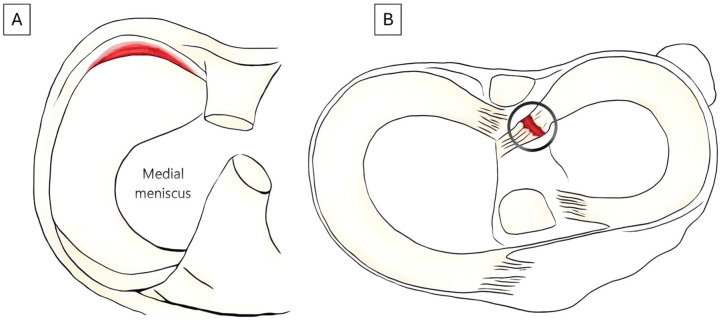
Illustration of a ramp lesion and lateral meniscus posterior root tear in the Norwegian Knee Ligament Register. (A) Rupture of the medial meniscus connection to the joint capsule. (B) A lateral posterior root tear is defined as an avulsion of the root or a significant radial tear less than 10 mm from the insertion.

*Intraoperative findings:* cartilage injuries (the International Cartilage Repair Society Classification System grade and size), graft choice for ACLR (patellar tendon, hamstring tendon, quadriceps tendon, other [including allograft]), and duration of surgery. Tear type was described separately for the medial and lateral menisci, and the surgeon could choose the one description that best described the intraoperative morphologic finding (radial, longitudinal, horizontal, anterior root tear, posterior root tear, bucket-handle tear, complex ruptures, or ramp lesion) (Figure 2). Treatment strategy and the number of sutures were also reported (surgeon could choose more than 1 option): partial resection, total resection, meniscus transplant surgery, all-inside suture, suture with cortical fixation, and/or other suture methods (including repairs through a posteromedial portal).

### Statistics

Descriptive statistics were used to give an overview of the patients’ demographics. Numeric variables were presented as means with standard deviations and categorical variables as numbers with percentages. Descriptive data between patients with and without meniscus injuries were compared—categorical variables were described using frequencies and further analyzed with the Pearson chi-square test and numeric variables (mean [SD]) tested with the Student *t* test or analysis of variance. Multivariate logistic analysis was performed to confirm the findings presented (Table 1). All tests were 2-sided, and the significance level was set to .05. All analyses were performed using SPSS version 29.0 (SPSS, Inc) and Stata 18.0 (StataCorp LLC). Sports participation at the time of injury was recorded, and the risk of concomitant meniscus injuries (to the ACL tear) for each sport was calculated.

**Table 1:** Patient Characteristics and Surgical Details in Isolated ACLR Versus ACLR With All Types of Meniscus Injuries Versus ACLR With LMPRT and Ramp Lesion*^*a*^*

Characteristic	Isolated ACLR	ACLR With Meniscus Injuries	*P* Value*^*b*^*	ACLR With LMPRT	*P* Value*^*b*^*	ACLR With Ramp Lesion	*P* Value*^*b*^*	Total
All knees, No.	1704	3605		531		415		5309
Male, No. (%)	747 (43.8)	2020 (56.0)	<.001	324 (61.0)	<.001	231 (55.7)	<.001	2767 (52.1)
Height, mean (SD), cm								
Male	179.9 (7.2)	180.0 (7.0)	.526	180.2 (6.8)	.548	180.7 (6.6)	.157	180 (7.0)
Female	166.9 (7.0)	167.3 (6.4)	.101	168.1 (6.6)	.016	167.5 (6.9)	.267	167.1 (6.6)
Weight, mean (SD), kg								
Male	81.9 (13.5)	83.7 (14.1)	.003	84.0 (13.8)	.021	82.4 (12.9)	.607	83.2 (14.0)
Female	68.4 (13.6)	69.4 (12.9)	.065	70.9 (12.8)	.019	67.6 (10.8)	.409	69.1 (13.2)
Age at ACLR, mean (SD), y	27.9 (10.5)	28.5 (10.8)	.041	29.4 (10.6)	.003	25.3 (8.9)	<.001	28.3 (10.7)
Age at injury, mean (SD), y	26.6 (10.3)	26.8 (10.6)	.498	28.1 (10.5)	.005	23.9 (8.7)	<.001	26.8 (10.5)
Time from injury to surgery, mean (SD), y	1.2 (2.3)	1.7 (3.2)	<.001	1.3 (2.6)	.287	1.4 (2.4)	.114	1.5 (2.9)
Previous surgery same knee, No. (%)	134 (7.9)	230 (6.4)	.108	34 (6.4)	.265	15 (3.6)	.002	364 (6.9)
Previous surgery contralateral knee, No. (%)	124 (7.3)	299 (8.3)	.201	43 (8.1)	.530	39 (9.4)	.146	423 (8.0)
Preoperative KOOS, No. (%)								
Symptoms	72 (17.4)	65.9 (19.3)	<.001	66.4 (17.8)	<.001	71.3 (18.8)	.564	67.9 (18.9)
Pain	74.5 (18.2)	70.1 (19.9)	<.001	70.9 (18.6)	.003	74.8 (18.8)	.862	71.5 (19.5)
ADL	82.8 (19.0)	77.4 (21.6)	<.001	77.2 (21.4)	<.001	82.0 (18.9)	.563	79.2 (20.9)
Sport	46.6 (27.7)	39.3 (28.3)	<.001	37.6 (27.1)	<.001	45.5 (27.6)	.597	41.7 (28.3)
QOL	37.9 (19.5)	33.7 (19.7)	<.001	33.6 (31.3)	<.001	35.6 (19.8)	.121	35.1 (19.7)
Level of activity,*^*b*^* No. (%)			.363		.746		.128	
Level 1	952 (57.5)	1953 (55.6)		295 (56.7)		254 (61.7)		2905 (56.2)
Level 2	353 (21.3)	753 (21.5)		108 (20.8)		84 (20.4)		1106 (21.4)
Level 3	312 (18.8)	694 (19.8)		100 (19.2)		60 (14.6)		1006 (19.5)
Level 4	40 (2.4)	110 (3.1)		17 (3.3)		14 (3.4)		150 (2.9)
Cartilage injury,*^*b*^* No. (%)			<.001		<.001		<.001	
ICRS 1-2	202 (11.9)	710 (19.7)		88 (16.6)		78 (18.8)		912 (17.2)
ICRS 3-4	88 (5.2)	320 (8.9)		66 (12.4)		22 (5.3)		408 (7.7)
None	1414 (83.0)	2575 (71.4)		377 (71.0)		315 (75.9)		3989 (75.1)
Type of graft,*^*b*^* No. (%)			.940		.422		.007	
BPTB	1375 (80.7)	2927 (81.2)		441 (83.1)		356 (85.8)		4302 (81.0)
Hamstring	289 (17.0)	586 (16.3)		76 (14.3)		44 (10.6)		875 (16.5)
Other	39 (2.4)	92 (2.5)		14 (2.6)		15 (3.6)		131 (2.4)
Duration of surgery, mean (SD), min	75.6 (23.2)	95.7 (31.7)	<.001	105.9 (33.9)	<.001	105.2 (30.0)	<.001	89.2 (30.7)

a ACLR, anterior cruciate ligament reconstruction; ADL, activities of daily living; BPTB, bone–patellar tendon–bone; ICRS, International Cartilage Repair Society Classification System; KOOS, Knee injury and Osteoarthritis Outcome Score; LMPRT, lateral meniscus posterior root tear; min, minutes; QOL, quality of life.

b Compared with isolated ACLR.

### Ethics

NKLR was approved by the Norwegian Data Inspectorate in 2004. Inclusion in the NKLR is based on written informed consent signed by the patient or the legal guardian (if the patient is <16 years of age). Therefore, this study needed no additional approval by the Regional Ethics Committee.

## Results

### Meniscus Tear Patterns in Primary ACLRs

Among the 5309 ACLR knees included in the study, 3605 knees (68%) had meniscus injuries (Figure 1). LMPRTs and ramp lesions were identified in 10.0% and 7.8% of the cohort, respectively (Table 1). A combination of both these meniscus injuries was present in 1.1% of knees. The most common type of lesion, however, was a longitudinal tear, observed in 1 or both menisci in 23% of knees. Bucket-handle tears were the second most common tear type, documented in 18.5% of knees (Table 2).

**Table 2:** Distribution of the Reported Meniscus Tear Types

Tear Type	Medial Meniscus Tears, No. (%)	Lateral Meniscus Tears, No. (%)
Radial tear	86 (3.4)	328 (15.4)
Longitudinal	715 (28.4)	626 (29.4)
Horizontal	99 (3.9)	99 (4.76)
Anterior root tear	6 (0.2)	12 (0.6)
Posterior root tear	59 (2.3)	531 (24.9)
Bucket handle	742 (29.5)	277 (13.0)
Complex rupture	362 (14.4)	216 (10.1)
Ramp lesion	415 (16.5)	3 (0.1)
Type not reported	32 (1.3)	39 (1.8)
Total	2516 (100)	2131 (100)

### Patient Characteristics

Of the patients with any meniscus injury, 56% were men. Additionally, 61% of patients with LPMRTs and 55.7% of ramp lesions were men. Even though 52% of patients in the entire ACLR cohort were men, these differences were significant. Patients with any meniscal injury had a higher body weight than those without (men, 83.7 vs 81.9 kg, *P* = .003; women, 69.4 vs 68.4 kg, *P* = .065). Those with ramp lesions were significantly younger at the time of injury (mean [SD], 23.9 [8.7] years) compared with those without meniscus injuries (mean [SD], 26.6 [10.3] years)—but also compared with those with an LMPRT (mean [SD] age, 28.1 [10.5] years). Furthermore, patients with meniscus injuries had lower preoperative KOOS scores across all subscales as compared with those with no meniscus injury (*P* < .001). This difference, however, was not seen for patients with ramp lesions (*P* = *ns*). The presence of cartilage injury was correlated (*P* = .001) with the presence of meniscus injury.

### Meniscus Injury Pattern Across Different Sports

Soccer was identified as the most common activity associated with ACL injuries (with concomitant meniscus injuries), accounting for 36.4% (n = 1932) of cases, followed by winter sports (alpine skiing, snowboarding, and skiing) at 18.5% (n = 984) and handball at 11.5% (n = 610). Table 3 displays the meniscus tear-type distribution across the most frequent sports performed at injury. Winter sports, such as cross-country skiing, snowboarding, and alpine skiing, exhibited the highest sports-specific incidence rates for both LMPRTs (14.3%) and ramp lesions (14.2%).

**Table 3:** Incidence of All Types of Meniscus Tears, LMPRTs, and Ramp Lesions Across Sports Reported Leading to the ACLR*^*a*^*

Sport	All Types of Meniscus Tears,*^*b*^* No. (%)	LMPRT,*^*c*^* No. (%)	Ramp Lesion,*^*d*^* No. (%)
Soccer	1338 (69.3)	207 (10.7)	192 (9.9)
Handball	398 (65.2)	64 (12.1)	50 (12.0)
Alpine/snowboard/ski	592 (60.2)	76 (14.3)	59 (14.2)
Basketball	76 (71.7)	6 (1.1)	8 (1.9)
Martial arts	67 (65.7)	9 (1.7)	4 (1.0)
Other team sports	110 (67.9)	19 (3.6)	16 (3.9)
Physical activities	357 (71.4)	60 (11.3)	31 (7.5)
Other	667 (73.1)	90 (16.9)	55 (13.7)

a ACLR, anterior cruciate ligament reconstruction; LMPRT, lateral meniscus posterior root tear.

b ACLR including all types of meniscus injuries.

c ACLR and LMPRT.

d ACLR and ramp lesion.

### Treatment of LMPRTs and Ramp Lesions

Nonoperative treatment was rarely chosen for LMPRT (5.6%) and ramp lesions (4.8%). The predominant treatment for LMPRT was transtibial suture with cortical fixation. For ramp lesions, the treatment strategies were split between an all-inside repair (n = 313) and other suturing techniques (including repairs through a posteromedial portal) (n = 108).

Across all repairs, an average of 3 sutures (or implants) were used per procedure. Table 4 provides further detailed information about the various reported treatment strategies.

**Table 4:** Treatment Strategy for Lateral Meniscus Posterior Root Tears and Ramp Lesions*^*a*^*

Treatment	Lateral Meniscus Posterior Root Tear (n = 531), No.	Ramp Lesion (n = 415), No.
Nonoperative treatment	30	20
Partial resection	43	5
Suture “all-inside”	47	313
Suture with cortical fixation	451	0
Other suture method*^*b*^*	8	108
Transplant	0	0
Total resection	0	0

a Surgeon could report multiple of the given treatments options per injury.

b Including repairs through a posteromedial portal.

## Discussion

In this first nationwide study reporting on the incidence and treatment of LMPRTs and ramp lesions during primary ACLRs, an incidence of 10.0% and 7.8%, respectively, was seen. The most frequent surgical treatment for LMPRT involved transtibial suture repair with cortical fixation, whereas ramp lesion repair was split between all-inside and other (commonly meaning a repair through a posteromedial portal) techniques. Strong associations were observed between male sex and meniscus injuries. Patients with ramp lesions were significantly younger than those without meniscus injuries and those in the LMPRT group. Across sports performed at the time of ACL tears, winter sports such as cross-country skiing, snowboarding, and alpine skiing demonstrated the highest sport-specific risk for LMPRTs and ramp lesions.

The current finding of a 68% overall incidence of meniscus injuries in primary ACLR is slightly higher than a report from Norway from 2005 to 2010 (49%)^22^ but also compared with previous reports from other international cohorts.^33,38,44^ There is no evidence that a change in the injury mechanism, through type (or degree) of sports participation, is responsible for an actual increased incidence of meniscus injuries overall. The main reason for the evident increase in reported meniscus injuries at ACLR could be a raised awareness of meniscus injuries, particularly LMPRTs and ramp lesions. Newfound knowledge about their role in maintaining normal knee biomechanics and the intimate relationship between meniscus dysfunction and the development of osteoarthritis—through changes in cartilage pressure—has likely changed our perception of how to handle these injuries. Norway has, by tradition, a high proportion of nonoperatively treated ACL injuries.^21^ This practice could potentially influence these figures, as there might be a selection bias toward more frequent surgery in patients with prevalent meniscus injuries caused by magnetic resonance imaging findings and more clinical symptoms leading them to seek surgical treatment—but also as a result of an increased risk of meniscus injuries during an expectant nonoperative treatment approach.^19^

The present study found a 10% rate of LMPRTs among 5309 primary ACLRs, performed by a range of surgeons across the country—representing a cross section of the clinical practices in Norway. Our findings are consistent with earlier studies reporting LMPRT rates ranging from 6.6% to 12%. Notably, Ahn et al^5^ documented a 7% occurrence of LMPRTs 15 years ago, and this incidence is corroborated by more recent reviews by Feuch et al^17^ and Wu et al.^52^

Ramp lesions were identified in 7.8% of all knees undergoing ACLR. In contrast, Thaunat et al^48^ reported a higher incidence of 15.5% in a cohort of 2156 patients over a 3-year period. Similar results were found by DePhillipo et al,^12^ Seil et al,^40^ Sonnery-Cottet et al,^45^ and Balazs et al,^6^ who all reported relatively high prevalence rates compared with our findings. Overall, the prevalence of meniscus ramp lesions diagnosed arthroscopically at the time of ACLR displays a wide range, from 9% to 42%.^6,12,31,44^ The lower incidence in the present study may be explained by different factors. Some authors display how ramp lesions can often be overlooked during arthroscopic examination and emphasize the need for looking at ramp lesions via a Gilquist portal or a separate posteromedial portal,^54^ and variation in the approach for visualizing the posterior meniscus is therefore held as a potential reason for this discrepancy. Other explanations can be related to the demographics of study populations or the greater uncertainty and disagreement regarding the precise definition of a ramp lesion. The average duration from injury to surgery in Norway exceeds 1 year, which means that small ramp lesions may have already healed by the time of ACLR.

Male sex and concomitant cartilage injuries were identified as general risk factors for all types of meniscus injuries in the current study. The sex difference may be explained by a lower ACL resilience in females, in whom the ACL ruptures at lower forces do not necessarily also damage secondary restraints like the menisci.^15,36^ Increased force during pivoting when injuring the ACL can lead to a combination of cartilage injuries, meniscus injuries, and ACL damage. LMPRT injuries were associated with lower preoperative KOOS values, concomitant medial meniscus injuries, a higher body mass index, and a slightly older age at the time of reconstruction. In contrast, the ramp lesion group was significantly younger—and the presence of this tear type did *not* yield a lower mean KOOS score. Previous studies have reported similar risk factors for LMPRTs and ramp lesions: age, male sex, and an association between medial meniscus injury and LMPRT and body mass index.^16,28,29,33,37,52^ The correlation between meniscus injuries and cartilage damage may suggest that higher impact or greater force at the time of injury could lead to more severe injuries. A novel finding of the current study is that the ramp lesion group, on average, was 4 years younger than the LMPRT group. Age <30 years has been described as a risk factor for both LMPRT and ramp lesions.^33,45^ This age disparity between the groups has not been highlighted in previous studies and should therefore be validated in other cohorts. Age is closely related to level of activity, and one could therefore speculate whether the differences in sports participation—and intensity of sports—could be a contributor to this difference.

This investigation examines the relationship between activity at the time of ACL injury and the incidence of LMPRT and ramp lesions. Among winter sports, such as cross-country skiing, snowboarding, and alpine skiing, the highest sports-specific incidence rates were observed for both LMPRTs (14.3%) and ramp lesions (14.2%). This finding may be explained by the high forces involved in trauma due to alpine skiing and snowboarding. In a previous study, where data from the NKLR were combined with the Kaiser Permanente ACLR Registry (2004-2011), the overall prevalence of meniscus injuries was reported across different sports: 51% in soccer, 43% in skiing, 63% in American football, 65% in basketball, and 45% in team handball.^23^ Kilcoyne et al,^27^ who examined 352 military cadets who underwent ACLR in 2012, found that wrestling displayed the highest risk of concomitant meniscus injury at 77%. Their findings on cumulative incidence in soccer (46%), skiing (43%), and handball (64%) showed lower rates than ours. As these latter 2 studies have less granular meniscus injury variables (yes/no), the association between sport and LMPRT and ramp lesions could not be examined. Sport-specific cohorts, like that by Farinelli et al,^14^ who investigated 40 consecutive elite soccer players who underwent ACLR by a single surgeon, found a high number of LMPRTs (25%) and ramp lesions (31%). Given the evident variability in meniscus tear rates across different sports, we cannot currently conclude that any one sport leads to a higher incidence of LMPRTs and ramp lesions than others.

Treatment options for LMPRT include nonoperative management, meniscectomy, partial meniscectomy, and root repairs.^35^ LMPRT repair during ACLR has overall been associated with good clinical outcomes.^41,49^ Shelbourne et al^42^ reported that at a mean 10-year follow-up of posterior lateral meniscus root tears left in situ, only mild lateral joint space narrowing was observed, and there were no significant differences in subjective or objective scores versus controls. Although clinical outcomes were not improved, this radiographic narrowing has been cited to support the repair of LMPRTs. In our study, including 531 knees with LMPRTs, the predominant treatment approach/suture technique involved using a transtibial suture with cortical fixation over a button or in an anchor. Several authors have described the safety of this technique.^10,35,54^ Zheng et al^53^ published a 2021 systematic review concluding that in root avulsions—or radial tears with tissue remnants of questionable quality—a transtibial pullout repair is recommended. Other techniques, using suture anchor repair, have theoretical advantages as they avoid the need for tibial bone tunnels, which could interfere with concomitant ligament reconstruction, and provide stable meniscus fixation closer to the joint line. However, optimal location and orientation of suture anchor placement have yet to be identified in the literature.^7^ Further, the use of an all-inside technique could perhaps be preferred in radial tears with a significant root remnant of adequate tissue quality.^35,53^ Ahn et al^5^ published a case series on side-to-side repair in such radial tears with good clinical results. Given that surgeons in our report could employ multiple options when reporting the mode of treatment, some cases also involved partial resection (8%), all-inside suturing (9%), and nonoperative management (5%). This reporting reflects the complexity of meniscus repair, where a customization of techniques is sometimes needed.

The treatment strategy for ramp lesions at the time of ACLR is still controversial.^32^ Bumberger et al,^9^ in their 2020 review, concluded that the current literature does not provide uniform generalized treatment recommendations for ramp lesions. In a randomized controlled trial from 2017, Liu et al^32^ compared treatment of stable, small- to medium-sized ramp lesions. While 1 group underwent repair, the other group only received trephination at the time of ACLR. The study concluded that those receiving trephination only achieved clinical outcomes comparable with surgical repair. In our study, nonoperative treatment was seen in only 5% of cases. Ramp lesion repair leads to a significant improvement in subjective knee scores, regardless of the specific fixation technique.^9^ In their review from 2020, Acosta et al^1^ advocate that the preferred approach for the repair of ramp lesions is the all-inside technique, while the least preferred technique for the repair of ramp lesions is the outside-in repair technique with a suture hook. On the other hand, 2 studies^17,26^ have suggested a lower failure rate and secondary meniscectomy with a suture hook repair through the posteromedial portal compared with an all-inside technique. There is a disagreement on the use of terminology to denote the different repair techniques: some use the term *all-inside* (traditionally denoting a suture from anterior to posterior with deployable implants) for the technique in which one uses an accessory posteromedial portal for instrumentation.^43,47^ Our findings with approximately three-fourths all-inside and one-fourth with other suturing techniques must therefore be interpreted with caution—the frequency of posterior medial portal usage might be higher than one-fourth.

### Strengths and Limitations

The participation of hospitals from both the private and the public sectors located across the whole country is a strength, as the numbers displayed are a representative cross-section of the whole nation. The NKLR contains data on the surgical treatment protocols but lacks information about preoperative magnetic resonance imaging findings, which could influence the surgeon's decision-making. The NKLR does not capture the incidence of meniscus tears in ACL injuries successfully treated nonoperatively. Variability in surgeon decision-making and differences in reported incidence and treatment approaches may also be affected by unclear or inconsistent definitions of meniscus injury types, as well as the high number of surgeons and hospitals involved in the registry. Further, we do not have detailed data on the subclassification of LMPRTs and ramp lesions that might likely affect the choice of treatment.^25,30,47^ This study does not include outcome data of these findings, and further research investigating clinical outcome, meniscus failure, and return to sport is needed.

The clinical implication of this inaugural descriptive national study is that all ACL surgeons should actively seek and anticipate the presence of both LMPRTs and ramp lesions (along with other types of meniscus tears) during ACLR. Furthermore, this study indicates that young males participating in winter sports have the highest risk of such injuries. A high suspicion of meniscus tears should therefore be held when consulting these patient groups.

## Conclusion

This first nationwide registry study identified a high incidence of concomitant meniscus tears in ACLR, with LMPRTs and ramp lesions at 10.0% and 7.8%, respectively. Patients with ramp lesions were considerably younger than both those with LMPRTs and those without meniscus injuries. Treatment for LMPRTs predominantly involved transtibial sutures with cortical fixation, while treatment for ramp lesions was divided between all-inside suturing techniques and other suturing methods (including repairs through a posteromedial portal).
